# Genetic association and functional implications of AhR gene polymorphism on preeclampsia

**DOI:** 10.3389/fcvm.2025.1567127

**Published:** 2025-10-15

**Authors:** Hong Jiang, Haiyun Meng, Yantuanjin Ma, Yuan Qian, Xuerong Chen, Lan Luo, Yuling Yang

**Affiliations:** ^1^ College of Basic Medical Sciences, Kunming Medical University, Kunming, Yunnan, China; ^2^ Clinical Laboratory, Jiangxi Province Hospital of Integrated Chinese and Western Medicine, Nanchang, Jiangxi, China; ^3^ Obstetrics Department, Yan'an Hospital of Kunming City, Kunming, Yunnan, China; ^4^ Institute of Biomedical Engineering, Kunming Medical University, Kunming, Yunnan, China; ^5^ Center of Clinical Precision Medicine and Diagnosis, The Affiliated Hospital of Yunnan University, Kunming, Yunnan, China

**Keywords:** preeclampsia, aryl hydrocarbon receptor, genetic polymorphism, angiogenesis, rs713150G

## Abstract

**Background:**

Preeclampsia is one of the main causes of increased maternal and infant mortality and morbidity during pregnancy. The aryl hydrocarbon receptor (AhR) is a ligand-activated transcription factor ubiquitously found in mammals that can directly or indirectly regulate various physiological processes when activated.

**Methods:**

In this case-control study, 74 patients with preeclampsia and 120 healthy pregnant controls were recruited. SNaPshot technology was used to detect genetic polymorphisms in four TagSNPs of the *AhR* gene. In addition, quantitative real-time PCR and western blotting were used to detect the expression of AhR in placental tissues from 21 patients with preeclampsia and 20 healthy controls. Subsequently, siRNA and drug treatment were used in vitro studies to knock down and inhibit AhR expression in human umbilical vein endothelial cells (HUVECs).

**Results:**

The allele frequency of rs713150G in the preeclampsia group was lower than that in the control group (OR = 0.467, 95% CI = 0.286-0.763; *P* = 0.002). Detection of AhR expression levels in placental tissue revealed that individuals who did not carry the rs713150G allele had lower expression of AhR in placental tissue than did those who carried the rs713150G allele, and lower AhR expression and nuclear translocation were positively correlated with the occurrence of preeclampsia. *In vitro* studies revealed that low expression of AhR in HUVECs suppressed the AhR signalling pathway, inhibited the expression of vascular endothelial growth factor A (VEGF-A), and inhibited the tube formation of HUVECs. And inhibition of AhR activity had a similar effect.

**Conclusion:**

The *AhR* gene polymorphism is associated with susceptibility of preeclampsia in the Chinese population and share in its pathogenesis as noncarriage of rs713150G leads to low expression of AhR in placental tissue that subsequently might participate in the development of preeclampsia by inhibiting placental angiogenesis.

## Introduction

Preeclampsia (PE) is a pregnancy-related condition characterized by hypertension and proteinuria, manifesting as hypertension, proteinuria, oedema, foetal growth restriction, etc., after 20 weeks of gestation (1). It is a common obstetric complication, with an incidence rate of 5%–8% (2). Worldwide, each year, it is responsible for over 500,000 fetal and neonatal deaths and over 70,000 maternal deaths (3), severely affects the health of mothers and infants, and is a major cause of increased maternal and perinatal foetal morbidity and mortality.

The pathogenesis of preeclampsia is still uncertain. The symptoms of preeclampsia can be relieved with the delivery of the placenta, so many scholars believe that the placenta plays a role in the development of preeclampsia. In normal pregnancy, placental angiogenesis and spiral artery remodeling provide sufficient blood perfusion to the placenta (4), and vascular endothelial growth factor (VEGF) plays a central role in angiogenesis (5). In preeclampsia, placental ischaemia and hypoxia are caused by “shallow placental implantation” and the extremely insufficient recasting of the uterine spiral artery, resulting in a series of symptoms due to the inability to exchange materials between the mother and foetus and the obstruction of foetal development (5). Research has shown that patients with preeclampsia have less blood flow in the placenta than do normal pregnant women (6) and a significantly smaller spiral artery diameter (4). In addition, the onset of preeclampsia tends to be familial, with a heritability of approximately 55% (7). Studies on the familial aggregation of pregnant women with preeclampsia and the concordance of preeclampsia occurring in monozygotic twins have also shown that there is genetic susceptibility to the development of the disease (8).

The aryl hydrocarbon receptor (AhR) is a ligand-activated transcription factor belonging to the basic helix-loop-helix (bHLH) Per–Arnt–Sim (PAS) transcription factor family. It is approximately 50 kb long and contains 11 exons located on chromosome 7p21.1. The *AhR* gene has multiple SNPs and is associated with the occurrence of various diseases. In the resting state, AhR is a cytoplasmic receptor protein that is activated by ligands and transferred to the nucleus to act as a transcription factor, directly or indirectly regulating various physiological processes. AhR is expressed in most tissues except skeletal muscle, with the highest expression levels in the liver, lungs, and placenta (9). AhR is expressed in the endothelial cells of villous large-blood vessels, the endothelial cells of umbilical cord veins/arteries and the decidua, with individual differences in expression (10). The human cytochrome P450 1A1 (*CYP1A1*) gene is one of the main target genes of AhR (11). CH223191, a specific antagonist of AhR, can effectively inhibit the nuclear translocation of AhR and the expression of the target gene *CYP1A1* (12). In this study, we investigated the correlation between *AhR* gene polymorphisms and preeclampsia, as well as the role of AhR in angiogenesis, to identify preeclampsia susceptibility genes conducive to the early diagnosis and prediction of this disease and to provide a valuable research basis for an in-depth exploration of the mechanisms of preeclampsia.

## Materials and methods

### Subjects

In this case‒control study, 194 pregnant women were recruited, including 74 patients with preeclampsia and 120 normal pregnant women. The samples collected for the study included the peripheral blood of pregnant women (preeclampsia: control = 74:120) and placental tissue after delivery (preeclampsia: control = 21:20). All samples were collected from pregnant women who gave birth at Yan'an Hospital affiliated to Kunming Medical University from June 2022 to October 2023. Preeclampsia was diagnosed using the criteria of the International Society for the Study of Hypertension in Pregnancy (ISSHP), as follows: gestational hypertension (clinic sBP 140 mmHg and/or dBP ≥90 mmHg) accompanied by one or more of the following new-onset conditions at ≥20 weeks' gestation: 1. Proteinuria; 2. Other maternal end-organ dysfunction, including neurological complications, pulmonary oedema, haematological complications, AKI and liver involvement; 3. Uteroplacental dysfunction (3). Subjects in the control group came from the same hospital and consisted of pregnant volunteer women with normal blood pressure, at least one prior pregnancy and no history of preeclampsia. Blood samples were collected by drawing 1 ml of venous blood from the pregnant woman and storing it in an EDTA-K_2_ anticoagulation tube at −80 °C. Tissue samples were collected within 5 min of delivery by cutting the placenta maternal surface into small pieces of approximately 1.0 cm × 1.0 cm × 1.0 cm under sterile conditions, avoiding necrotic, hemorrhagic and calcified areas. The tissue samples were rinsed thoroughly with PBS and stored at −80 °C. All participants signed an informed consent form for the collection of peripheral blood and placental tissue samples. The study was approved by the Medical Ethics Committee of Kunming Medical University (Approval No. KMMU2022MEC049).

### Genotyping of AhR SNPs

Genomic DNA was extracted from blood samples using the DNeasy Blood & Tissue Kit™ (69506, QIAGEN) according to the manufacturer's instructions. The obtained DNA was stored at −20 °C until analysis.

The required data were downloaded from the 1,000 Genomes Browser (13) (https://www.ncbi.nlm.nih.gov/variation/tools/1000genomes/). TagSNPs were selected using HaploView4.2 software (14) (the upstream and downstream range settings were 2K, MAF ≥ 0.05, *R*^2^ ≥ 0.8). Five TagSNPs for the *AhR* gene were obtained (Supplementary Table S1). The SNaPshot method was used to analyse the polymorphisms of the SNP sites. The primers used in our study are shown in Supplementary Table S2. For genotype frequency analysis, dominant and recessive models were specified *a priori*.

### Quantitative real-time PCR

Total RNA was extracted from placental samples utilizing the TRIzol method. The RNA from each sample was reverse transcribed to cDNA using reverse transcription kit (RR047A, Takara). qRT‒PCR was performed with a BlastTaq™ 2×qpcr MasterMix Kit (Applied Biological Materials Inc., Canada) and a QuantStudio 6 real-time PCR system under the following thermal cycle conditions: 42 °C for 20 min and 95 °C for 3 min, 40 cycles of 15 s at 95 °C and 1 min at 60 °C, 15 s at 95 °C, 1 min at 60 °C and 15 s at 95 °C. Relative fold changes in mRNA expression were calculated via the 2-ΔΔCt method. GraphPad Prism 6 was used to create graphs. The primer sequences are shown in Supplementary Table S3.

### Western blotting

Total protein was extracted with protein lysis buffer, and nuclear protein was extracted with a nuclear protein extraction kit (BB-3166, BestBio). The antibodies used for WB were anti-Ah receptor antibody (sc-133088, Santa Cruz Biotechnology), anti- Lamin B1 monoclonal antibody (66095-1-Ig, Proteintech), anti-CYP1A1 polyclonal antibody (13241-1-AP, Proteintech), anti-VEGF-A monoclonal antibody (66828-1-Ig, Proteintech), β-actin monoclonal antibody (AF0003, Beyotime Biotechnology) and HRP-conjugated Affinipure goat anti-mouse IgG (H + L) (SA00001-1, Proteintech).

### HUVEC culture

Human umbilical vein endothelial cells (HUVECs, GNHu39) were purchased from the cell bank of the Chinese Academy of Sciences. HUVECs were cultured in RPMI-1640 medium supplemented with 10% FBS (CO4001, VivaCell) and 1% penicillin‒streptomycin (C3421‒0100, VivaCell). The cells were incubated at 37 °C in a 5% CO_2_ atmosphere. HUVECs from the third to sixth passages were used in the experiments. The cells were seeded in 6-well plates, and subsequent experiments were conducted when they reached 50%–70% confluency.

### Knockdown of AhR using small interfering RNA

The siRNA sequence was designed on the basis of the sequence provided by NCBI GenBank (15) (Supplementary Table S4). Cells were transfected with siRNA at a concentration of 40 nmol/well in DMEM (C3113, VivaCell) via PEI transfection reagent (24765-1, Polysciences) according to the manufacturer's instructions. After 4 h, the medium was replaced with normal HUVEC medium, and the cells were incubated for 24 h in a suitable environment. The mRNA and protein expression levels of AhR and its target gene CYP1A1 were detected via the methods described in Sections 2.3 and 2.4 to evaluate the efficiency of siRNA interference.

### Inhibition of AhR activity using CH223191

An appropriate amount of DMSO solution was added to CH223191 powder (HY-12684, MedChemExpress) to formulate a 10 mM CH223191 stock solution. The optimal time and concentration of CH223191 were detected via a CCK8 assay (PF00004, Proteintech) according to the manufacturer's instructions. The protein expression levels of nuclear AhR, total AhR and CYP1A1 were detected via the methods described in Section 2.4 to evaluate the efficiency of inhibiting AhR activity.

### Tube formation assay

A 96-well plate was placed on ice, and 50 μl of Matrigel (356234, BD) was coated on each well. After incubation at 37 °C for 30 min, 5 × 10^4^ HUVECs in 100 μl of the logarithmic growth phase were seeded in each well and cultured at 37 °C and 5% CO_2_. Photos of capillary-like tubes were captured by an inverted microscope (Axio Observer.Z1, ZEISS, Germany) 4 h after seeding. The junctions, total length and total branching length of the tubes were quantified using ImageJ.

### Scratch assay

HUVECs (1 × 10^5^) in 1 ml of extract were seeded in 12-well plates. After attachment, the cells were transfected or dosed according to the experimental requirements and cultured at 37 °C and 5% CO_2_. When the cells reached 90% confluence, a scratch was made in the centre of the well using a 10 μl tip. Images were captured at 0 and 24 h using an inverted microscope (Axio Observer.Z1, ZEISS, Germany). The migration area was quantified using ImageJ.

### Statistical analysis

For each SNP, SHEsis software (16) was used to evaluate the Hardy‒Weinberg equilibrium, the chi-square test or Fisher's exact test was used to compare gene distributions among groups, and multivariate logistic regression was employed to calculate odds ratios (ORs) and 95% confidence intervals (CIs). The allelic associations between the preeclampsia group and the control group were evaluated. For continuous variables, the Shapiro‒Wilk test was used to test normality. Normally distributed continuous variables are expressed as the mean ± standard deviation, and nonnormally distributed variables are expressed as the median and interquartile range (IQR). A *t* test was used to compare measured data between the groups. The *X*^2^ test was used to compare count data between groups. The GPower3.1.9.7 was used to calculate *post-hoc* power for SNP. ImageJ (17) was used to analyse the greyscale values of the images. SPSS 23.0 was used to perform one-way ANOVA. GraphPad Prism 6 was used to create figures. *P* < 0.05 was considered to indicate statistical significance.

## Results

### Clinical characteristics of pregnant women in the preeclampsia and control groups

As shown in Table 1, no significant differences in age and BMI were detected between the two groups (*p* > 0.05). However, there were significant differences in gestational age, primiparity, previous miscarriages and caesarean section between the two groups (*p* < 0.05).

**Table 1:** Clinical characteristics of pregnant women in the preeclampsia and control groups.

Variable	Preeclampsia (*n* = 74)	Control (*n* = 120)	*t/χ^2^*	*P*
Age (years)	35.176 ± 4.492	33.783 ± 5.567	1.911	0.058
BMI (kg/m^2^)	23.704 ± 3.614	24.491 ± 2.854	1.593	0.114
Gestational age (weeks)	38.365 ± 2.079	37.788 ± 1.621	2.039	**0**.**044**
Primiparity (%)	43.243	15.000	19.086	**<0**.**001**
Previous miscarriages (%)	70.270	50.833	7.111	**0**.**008**
Caesarean section (%)	56.757	37.500	6.862	**0**.**009**

Bold values indicate statistical significance (*P* < 0.05).

### Correlation between *AhR* TagSNPs and preeclampsia

#### Hardy–Weinberg equilibrium test for AhR gene TagSNPs

In this study, we tested 5 TagSNPs for the *AhR* gene in all blood samples. The HWE-P of rs7796976 was less than 0.05, and the remaining 4 loci passed the test for Hardy–Weinberg equilibrium (Supplementary Table S5). The rs7796976 locus was not included in the subsequent studies.

#### Allele frequencies in the preeclampsia and control groups

Among the 4 TagSNPs in the *AHR* gene passed the HWE test, the frequency of rs713150G in the preeclampsia group was lower than that in the control group (OR = 0.467, 95% CI = 0.286–0.763; *P* = 0.002). No significant differences in allele frequency for the other loci in the *AhR* gene were observed between the two groups (Table 2). With the actual sample size (total alleles) *N* = 388, *α* = 0.05, and the observed effect size Phi *φ* = 0.16, *post-hoc* power for rs713150 was 0.87.

**Table 2:** Distributions of allele frequencies in the preeclampsia and control groups.

SNPs	Alleles	Preeclampsia		Control		OR (95%CI)	*X^2^*	*P*
		*n*	%	*n*	%			
rs2066853	G	99	66.89%	170	70.83%	0.832 (0.535–1.293)	0.413	0.669
	A	49	33.11%	70	29.17%			
rs2158041	T	20	13.51%	49	20.42%	0.609 (0.346–1.073)	2.984	0.084
	C	128	86.49%	191	79.58%			
rs713150	G	28	18.92%	80	33.33%	0.467 (0.286–0.763)	9.469	**0**.**002**
	C	120	81.08%	160	66.67%			
rs10249788	C	105	70.95%	179	74.58%	0.832 (0.526–1.316)	0.617	0.479
	T	43	29.05%	61	25.42%			

#### Genotype frequencies in the preeclampsia and control groups

For rs713150, the alleles are C and G, and the G allele is the minor allele. The CC, GC and GG genotype frequencies of the preeclampsia group were 64.86%, 32.43%, and 2.7%, respectively, and those of the control group were 44.17%, 45.0%, and 10.83%, respectively (*P* = 0.008). The G allele, which has a lower frequency in the population, is considered a mutant gene. In the dominant model, those carrying this mutation were less likely to develop preeclampsia (GG + GC vs. CC, OR = 0.428, 95% CI = 0.236–0.779; *P* = 0.018). In the recessive model, this difference was not significant (GG vs. GC + CC). A significant association was observed only under the dominant model. Therefore, we suggest that if the rs713150G is associated with the development of preeclampsia, it likely follows a dominant model. No significant differences in genotype frequencies for the remaining detected TagSNPs were observed between the two groups (Table 3).

**Table 3:** Distributions of genotype frequencies in the preeclampsia and control groups.

SNPs	Preeclampsia (*n* = 74)			Control (*n* = 120)			*P*	AB + BB vs. AA		BB vs. AA + AB	
	AA	AB	BB	AA	AB	BB		OR (95% CI)	*P*	OR (95% CI)	*P*
rs2066853	29 (0.392)	41 (0.554)	4 (0.054)	59 (0.492)	52 (0.433)	9 (0.075)	0.259	1.501 (0.834–2.702)	0.185	0.705 (0.209–2.376)	0.769
rs2158041	54 (0.730)	20 (0.270)	0.000	75 (0.625)	41 (0.342)	4 (0.033)	0.233	0.617 (0.328–1.162)	0.133	1.034 (1.001–1.069)	0.144
rs713150	48 (0.649)	24 (0.324)	2 (0.027)	53 (0.442)	54 (0.45)	13 (0.108)	**0**.**008**	0.428 (0.236–0.779)	**0**.**005**	0.229 (0.050–1.044)	0.052
rs10249788	37 (0.5)	31 (0.419)	6 (0.081)	72 (0.6)	35 (0.292)	13 (0.108)	0.188	1.500 (0.837–2.690)	0.183	0.726(0.263–2.002)	0.625

A, major allele; B, minor allele.

Bold values indicate statistical significance (*P* < 0.05).

### Correlation between placental AhR expression levels and preeclampsia

**Figure 1:**
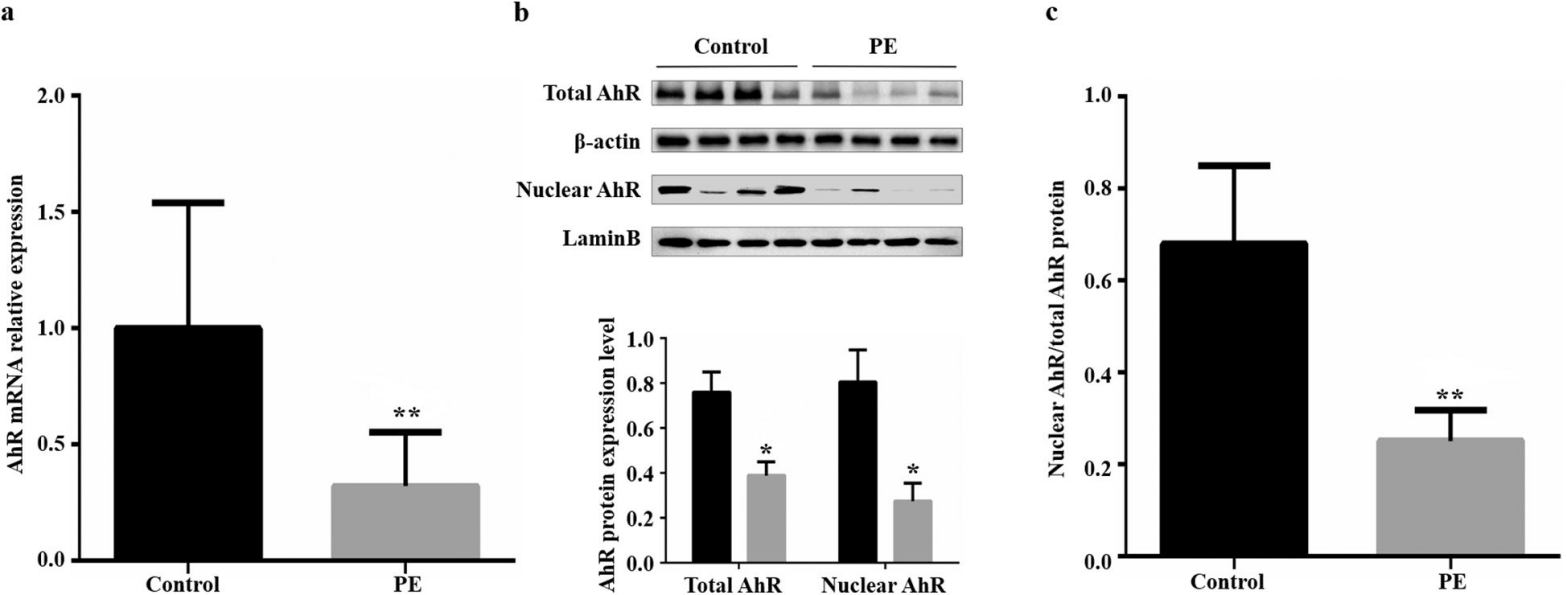
Placental AhR mRNA and protein expression in the control and preeclampsia groups. **(a)** Placental AhR mRNA expression; β-actin was used as an internal control. **(b)** Total and nuclear placental AhR protein expression; β-actin was used as the internal control for total protein, and LaminB was used as the internal control for nuclear protein. **(c)** Ratio of nuclear to total placental AhR protein. PE, preeclampsia; AhR, aryl hydrocarbon receptor. *P < 0.05, **P < 0.01.

The mRNA and protein expression levels of AhR were examined in all placental tissues. Compared with those from normal pregnant women, placental tissues from women with preeclampsia presented significant decreases in AhR mRNA expression (0.68, Figure 1a), total AhR protein expression (0.44, Figure 1b) and nuclear AhR protein expression (0.66, Figure 1b). We also compared the ratio of nuclear AhR protein to total AhR protein in placental tissues between the two groups, and the ratio was significantly lower in the preeclampsia group (control vs. preeclampsia: 0.680 ± 0.085 vs. 0.251 ± 0.034, *t* = 4.681, *P* = 0.003; Figure 1c). The inefficiency of nuclear transfer was associated with the inhibition of AhR.

### Correlation between the rs713150 polymorphism and AhR protein expression

The protein expression levels of AhR corresponding to different genotypes of the *AhR* gene rs713150 were analysed in all placental tissues. Based on the results of the genetic model study, analyses were conducted under the dominant model. The expression level of AhR with CC genotype was significantly lower than that with CG + GG genotype (CC vs. CG + GG: 0.152 ± 0.083 vs. 0.332 ± 0.297, *t* = −2.594, *P* = 0.016), suggesting that individuals who do not carry rs713150G have lower AhR protein expression. This corresponds with the results obtained in our aforementioned study: Compared with control group, the frequency of rs713150G was lower in the preeclampsia group, and the expression level of AhR in the placenta was also significantly reduced.

### Correlation between AhR and the angiogenic capacity of HUVECs

Previous studies have reported that AhR may be important in the regulation of vascular development (18–21). Our previous study revealed decreased AhR expression and nuclear translocation in the placentas of women with preeclampsia. This part of the study focused on the impact of inhibiting the AhR signalling pathway on angiogenesis.

#### Knockdown of AhR expression and its effect on angiogenesis in HUVECs

**Figure 2:**
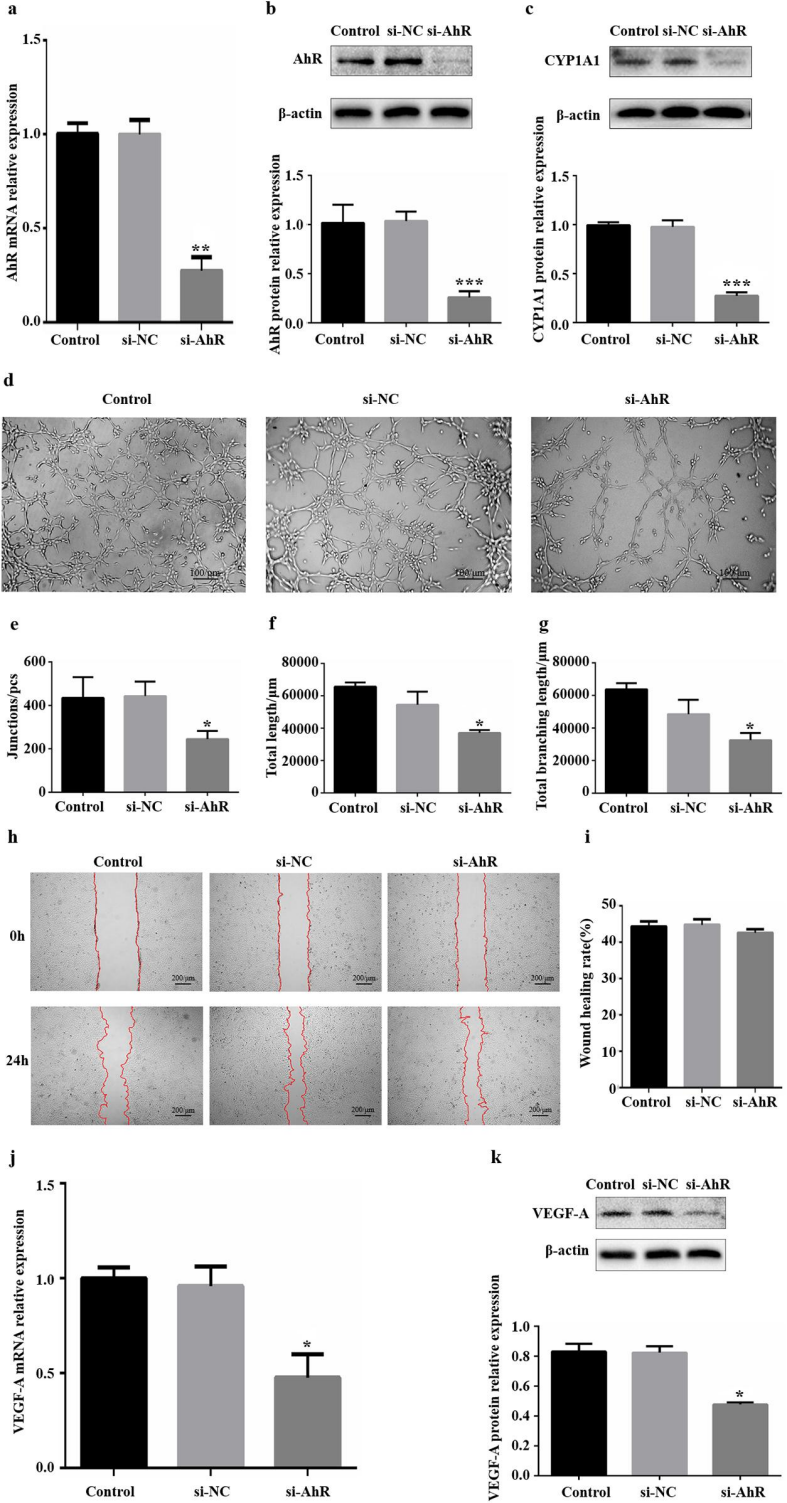
Influence of downregulation of AhR expression on angiogenesis *in vitro*. **(a)** Significant knockdown of *AhR* mRNA expression via siAhR. **(b)** Significant knockdown of AhR protein expression via siAhR. **(c)** CYP1A1 protein expression levels were significantly reduced in HUVECs that were transfected with siAhR for 24 h. **(d)** Images of HUVEC tube formation on Matrigel at 4 h. **(e)** Quantification of junctions (*n* = 3). **(f)** Quantification of total length (*n* = 3). **(g)** Quantification of total branching length (*n* = 3). **(h)** Images of HUVEC migration at 0 and 24 h; the red lines indicate cell migration fronts. **(i)** Quantification of the relative migration area. **(j)** Levels of VEGF-A mRNA expression after transfection with siRNA. **(k)** Levels of VEGF-A protein expression after transfection with siRNA. AhR, aryl hydrocarbon receptor; CYP1A1, cytochrome P450 1A1. *P < 0.05, **P < 0.01, ***P < 0.001.

HUVECs were transfected with siRNA specific for AhR (siAhR). The expression levels of AhR and its target gene CYP1A1 were detected by WB and RT‒qPCR to evaluate the efficiency of siRNA interference. Both AhR expression (Figures 2a,b) and CYP1A1 expression (Figure 2c) were significantly lower in the si-AhR group than in the si-NC group. These results suggest that the AhR-specific siRNA sequence effectively inhibited the expression of AhR in HUVECs, thereby suppressing AhR signalling.

Following the transfection of HUVECs with siAhR for 24 h, tube formation, migration and the expression of the angiogenic factor VEGF-A were used as important indicators of the angiogenic capacity of HUVECs *in vitro*. *in vitro* HUVEC tube formation assays revealed that fewer tubes formed in the si-AhR group than in the si-NC group (Figure 2d), indicating that vascularization inhibited by reduced level of AhR protein expression. Further analysis via ImageJ revealed that all three core indicators of tubular formation parameters were significantly reduced in the si-AhR group, with fewer intersections and shorter total tubular and branch lengths (Figure 2e–g, Supplementary Table S6). *in vitro* scratch assays revealed that there was no statistically significant difference in the relative migration area of HUVECs in the si-AhR group compared with the si-NC group (Figures 2h,i). The RT‒qPCR results revealed lower levels of VEGF-A mRNA transcripts (0.52) in the si-AhR group than in the control group (Figure 2j). In accordance with the RT‒qPCR results, downregulated levels of VEGF-A protein expression (0.43) were detected via western blotting in the si-AhR group (Figure 2k). Therefore, these findings indicate that reducing the expression of AhR caused a significant reduction in the tube-forming ability and a significant decrease in the expression of VEGF-A in HUVECs but had no significant effect on their migration ability.

#### Inhibition of AhR activity and its effect on angiogenesis in HUVECs

**Figure 3:**
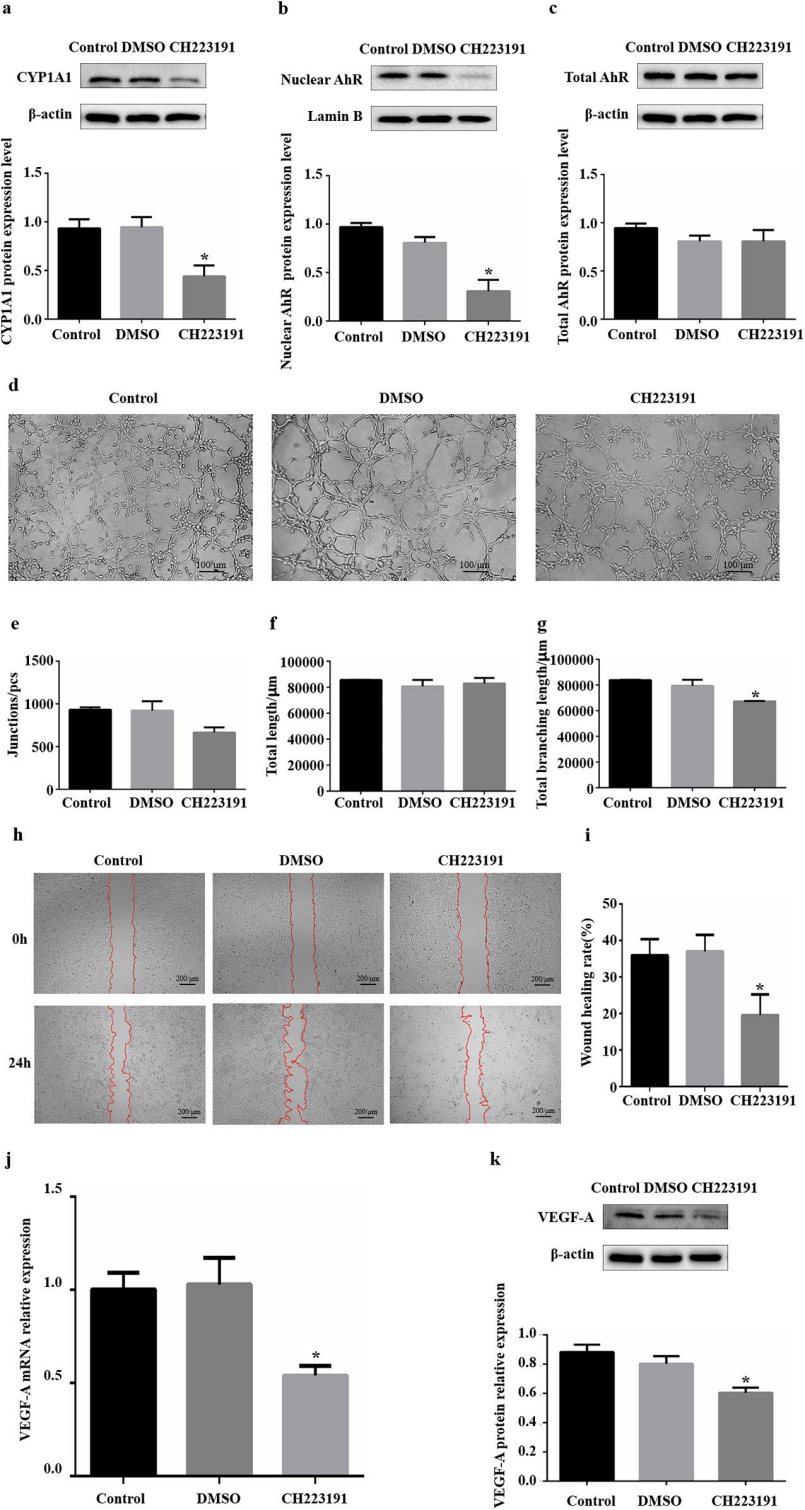
Influence of inhibiting AhR activity on angiogenesis *in vitro*. **(a)** CYP1A1 protein levels were significantly reduced in HUVECs after treatment with CH223191. **(b)** Nuclear AhR protein levels were significantly reduced in HUVECs after treatment with CH223191. **(c)** Total AhR protein levels were not significantly changed in HUVECs after treatment with CH223191. **(d)** Images of HUVEC tube formation on Matrigel at 4 h. **(e)** Quantification of junctions (*n* = 3). **(f)** Quantification of total length (*n* = 3). **(g)** Quantification of total branching length (*n* = 3). **(h)** Images of HUVEC migration at 0 and 24 h. The red lines indicate cell migration fronts. **(i)** Quantification of the relative migration area. **(j)** Levels of VEGF-A mRNA expression after CH223191 addition. **(k)** Levels of VEGF-A protein expression after CH223191 addition. AhR, aryl hydrocarbon receptor; CYP1A1, cytochrome P450 1A1. *P < 0.05.

CH223191 is an effective and specific antagonist of AhR that can inhibit the nuclear transfer of AhR and its binding to DNA. HUVECs were treated with 4 μM CH223191 for 24 h. The protein expression levels of CYP1A1, nuclear AhR and total AhR were detected via WB to evaluate the inhibitory efficiency of CH223191. Compared with those in the DMSO group, the CYP1A1 protein expression levels in the CH223191 group decreased (Figure 3a), the nuclear AhR protein expression levels decreased significantly, and the total AhR protein expression level was unchanged (Figures 3b,c). These results suggest that the CH223191 concentration and duration of treatment effectively inhibited the nuclear translocation of AhR in HUVECs, thereby suppressing AhR signalling.

Following the treatment of HUVECs with CH223191 for 24 h, tube formation, migration and VEGF-A expression were used as important indicators of the angiogenic capacity of HUVECs *in vitro*. *in vitro* HUVEC tube formation assays revealed that the formation of fragmented tubes in the CH223191 group was somewhat affected compared with that in the DMSO group (Figure 3d). Further analysis via ImageJ revealed that among the three core parameters of tubular formation, only the total branch length decreased significantly, and there were no significant differences in the other parameters (Figures 3e–g, Supplementary Table S6). *in vitro* scratch assays revealed that the relative migration area of HUVECs in the CH223191 group decreased by approximately 47% compared with that in the DMSO group (Figures 3h,i). The RT‒qPCR results revealed reduced levels of VEGF-A mRNA transcripts (0.46) in the CH223191 group compared with those in the DMSO group (Figure 3j). In accordance with the RT‒qPCR results, downregulated levels of VEGF-A protein expression (0.32) were detected via western blotting in the CH223191 group (Figure 3k). Therefore, these findings indicate that inhibiting AhR nuclear transfer significantly reduced the migration ability of HUVECs, decreased their tube-forming ability to a certain extent, and significantly decreased their expression of VEGF-A.

## Discussion

The development of preeclampsia is associated with insufficient remodeling of uterine spiral arterioles; thus, genes associated with angiogenesis or maintenance are likely susceptibility genes for preeclampsia. AhR was originally discovered in toxicology research as a mediator of toxicity caused by exogenous substances (22). In the past decade, researchers have become increasingly aware that endogenous activation of AhR has important effects on many normal physiological processes, such as mediating the proliferation of vascular cells in the body (18). There are many disease-associated SNP polymorphisms in the *AhR* gene. The occurrence of Crohn's disease was found to be associated with a mutation at the *AhR* gene rs2158041 in the Chinese population (23), whereas xeroderma (24), rheumatoid arthritis (25), glioma (26), and vitiligo (27) have been associated with polymorphisms in the SNPs of the AhR gene.

In this case‒control study, 74 patients with preeclampsia and 120 normal pregnant women were recruited. We first explored the associations of 4 TagSNPs in the AhR gene with preeclampsia. These 4 TagSNPs were located in the promoter region (rs10249788), exon region (rs2066853), and intron region (rs2158041 and rs713150), respectively. Our results showed that the genotype frequency and allele frequency of rs713150 were significantly different between the two groups and were likely to be inherited in a dominant model in the population. Further analysis revealed that the rs713150G allele frequency (MAF) in the preeclampsia group was significantly lower than that in the control group, and the OR was less than 1, indicating that this locus may be a protective factor for disease development. Therefore, we suggest that *AhR* gene polymorphism is associated with the development of preeclampsia and that rs713150G is a protective factor against disease development. By exploiting linkage disequilibrium in the genome, we selected tag SNPs that capture most variation within each LD block. Still, the issue of incomplete coverage remains unavoidable. This consequently introduces the possibility of missing other associated variants. The genetic mechanism of preeclampsia is extremely complex and remains to be fully elucidated. Further studies with larger sample sizes, multi-ethnic cohorts, and a greater number of SNP loci are still warranted.

We subsequently detected the mRNA and protein expression levels of AhR in the placenta maternal surface from patients with preeclampsia and normal pregnant women. The results revealed that the relative expression levels of AhR mRNA, total AhR protein, and nuclear AhR protein in the placental samples from women with preeclampsia were significantly lower than those in placental samples from normal pregnant women. Moreover, the ratio of nuclear AhR to total AhR in the placental samples from women with preeclampsia was significantly lower than that in placental samples from normal pregnant women. AhR, as a transcriptional activator, when activated upon binding to a ligand, enters the nucleus to regulate target gene transcription. That is, AhR only has the opportunity to perform its function upon entering the nucleus. Our results suggest that the development of preeclampsia is associated not only with reduced expression of AhR in the placenta but also with reduced efficiency of AhR activation.

Our study on the correlation between the AhR genotype and its molecular expression level revealed that the rs713150 polymorphism of the AhR gene is associated with the level of AhR protein expression. Individuals carrying the rs713150G allele have higher expression levels of AhR protein in the placenta. rs713150 is located in the intron region of the gene. Although introns are excised during mRNA processing, introns have been found to increase gene expression (28) and regulate selective splicing, mRNA transport, chromosome assembly, and other functions (29). Recent studies have shown that SNPs polymorphisms in introns are associated with various diseases (30, 31). Some studies have shown that intron regions also contain genes encoding proteins, and at least 158 protein-coding genes have been found in introns in humans (32). Mutations in introns may lead to changes in protein expression, structure, and stability, and even phenotypes (33). Therefore, we speculate that *AhR* gene polymorphisms may play an important regulatory role in gene expression and that rs713150 mutations may affect alternative splicing of genes, etc., in turn affecting the protein expression level of AhR.

We downregulated the AhR signalling pathway *in vitro* by knocking down AhR expression and inhibiting AhR activation, which resulted in inhibit the angiogenesis-related ability of HUVECs. Notably, the downregulation of AhR expression by siRNA affected mainly the tube formation ability of cells, whereas the inhibition of AhR activation by the inhibitor affected mainly the migration ability of cells. Knocking down protein expression with siRNA can controllably reduce the protein expression level by more than 70%, resulting in relatively clear results. For the tube-forming ability of HUVECs, AhR content is likely to play a major role; thus, downregulating the expression of AhR can significantly inhibit tube formation. However, the target of inhibitor is often not unique, the final effect of inhibitor depends on the co-regulation of multiple pathways. Thus, AhR activity may be one of the factors affecting HUVEC migration.

## Conclusion

*AhR* gene polymorphisms are associated with the development of preeclampsia in the Chinese population. The *AhR* gene is a susceptibility gene for the disease, and rs713150G is a protective factor for the development of the disease. Compared with individuals who carried the G allele, individuals who did not carry the G allele present lower expression of AhR in the placenta, and lower AhR expression is positively correlated with the development of preeclampsia. Low expression or inhibition of AhR activity inhibits the AhR signalling pathway and may participate in the development of preeclampsia by inhibiting angiogenesis. These data improve our understanding of the pathogenesis of preeclampsia.

## Data Availability

The raw data supporting the conclusions of this article will be made available by the authors, without undue reservation.
