# Supplementary material for: Genetic association and functional implications of AhR gene polymorphism on preeclampsia
Source: Front Cardiovasc Med. 2025 Oct 15;12:1567127. doi: 10.3389/fcvm.2025.1567127 (PMC12568678; doi:10.3389/fcvm.2025.1567127)
Supplement: Supplementary file 1 [file Datasheet1.pdf]

SNPs	Major Allele	Minor Allele	Chromosome Position	Localization	Function
rs10249788	C	T	chr7:17298523	promoter	Non Coding Transcript Variant
rs2158041	C	T	chr7:17328796	intron	Intron Variant
rs713150	C	G	chr7:17300533	intron	Intron Variant
rs2066853	G	A	chr7:17339486	exon	Missense Variant
rs7796976	A	G	chr7:17298806	5’UTR	5 Prime UTR Variant

Supplementary Table S1. Basic information on AhR gene SNPs

SNPs	Amplification				Extension	
	Forward primer	Reverse primer	Tm (℃)	Product (bp)	Primer	Direction
rs10249788	ATGCACGAAGATGGCTACCG	TAAATCCAGGGTGCTTCCCG	60	372	TTTTTTTTTTTTTTTTTTTTTTTTTGCCCATCTGGATTCCATTCC	R
rs2158041	GAGCCCCATTGTCAGATATTCTG	GACTTTGTGGTAGCAGTAGTAGC	60	323	TTTTTTTTTTTTTTTTTTTTTTTTTTTTTGCCAACCATTACACAATTTCC	R
rs713150	CAGACCAAATCAGAAGTGTGCA	TGTCTCAAACTGCCAAGGTAGA	60	301	TTTTTTTTTTTTCCTCTGTTAATGCCAATTCATAAGTATA	R
rs2066853	GAACTCATTTGCTGGAGGTCAC	AGGTGTTCCTGTACCATACAGC	60	291	TTTTTTTTTTTTTTTTTTTTTTAGGCATTGATTTTGAAGACATCA	F
rs7796976	CTCTGTTCCGAGAGCGTGC	GGAGCCTGGGCCGTCTATT	61	252	TTTTTTTTTTTTTTTTTTTTTTTTTTTTTTTCGGGAAGCACCCTGGATTT	F

Supplementary Table S2. Primers of SNPs

Gene	Primer		Tm (℃)	Product (bp)
AhR	F	GTCGTCTAAGGTGTCTGCTGGA	61.71	137

	R	CGCAAACAAAGCCAACTGAGGTG	63.4	
CYP1A1	F	TCGGCCACGGAGTTTCTTC	60.01	141
	R	GGTCAGCATGTGCCCAATCA	60.97	
VEGF-A	F	AGGGCAGAATCATCACGAAGT	59.44	75
	R	AGGGTCTCGATTGGATGGCA	60.98	
β-actin	F	CATGTACGTTGCTATCCAGGC	59.13	250
	R	CTCCTTAATGTCACGCACGAT	58.46	

Supplementary Table S3. Primers used in quantitative real-time PCR

siRNA	Sense strand	Antisense strand
CY3 siRNA	UUCUCCGAACGUGUCACGU TT	ACGUGACACGUUCGGAGAA TT
siRNA NC	UUCUCCGAACGUGUCACGU TT	ACGUGACACGUUCGGAGAA TT
GAPDH	UAAAGUACCCUGUGCUCAA TT	UUGAGCACAGGGUACUUUA TT
siRNA AhR	CGGAUGAAAUCCUGACGUA TT	UACGUCAGGAUUUCAUCCG TT

Supplementary Table S4. siRNA sequences

SNPs	AA	AB	BB	Total	χ^2	HWE-*P*
rs2066853	88(0.454)	93(0.479)	13(0.067)	194.000	3.140	0.207
rs2158041	129(0.665)	61(0.314)	4(0.021)	194.000	1.100	0.294
rs713150	101(0.521)	78(0.402)	15(0.077)	194.000	<0.001	0.999
rs10249788	109(0.562)	66(0.340)	19(0.098)	194.000	3.431	0.179
rs7796976	20(0.103)	174(0.897)	0(0.000)	194.000	89.239	**<0.001**

Supplementary Table S5. Hardy–Weinberg equilibrium test for each SNP. A: Major allele, B: minor allele

Group		Junctions (point)	Total length (μm)	Total branching length (μm)
Knockdown of AhR	Control	494.70 ± 158.80	$65.72\times10^3 \pm 1.46\times10^3$	$63.76\times103 \pm 2.20\times10^3$
	si-NC	443.00 ± 38.63	$54.54\times10^3 \pm 4.69\times10^3$	$48.54\times103 \pm 5.09\times10^3$
	si-AhR	**262.00 ± 41.68 ***	**$37.08\times10^3 \pm 1.06\times10^3$ ***	**$32.54\times103 \pm 2.52\times10^3$ ***
Addition of CH223191	Control	926.50 ± 7.50	$85.63\times10^3 \pm 0.14\times10^3$	$83.99\times10^3 \pm 0.28\times10^3$
	DMSO	922.00 ± 64.16	$80.69\times10^3 \pm 2.90\times10^3$	$79.49\times10^3 \pm 2.71\times10^3$
	CH223191	665.50 ± 41.50	$83.08\times10^3 \pm 2.89\times10^3$	**$67.41\times10^3 \pm 0.14\times10^{3*}$**

Supplementary Table S6. Quantification of tubule formation parameters in HUVECs. **P*<0.05 vs. si-NC group / DMSO group.
